# Adhesion Properties, Biofilm Forming Potential, and Susceptibility to Disinfectants of Contaminant Wine Yeasts

**DOI:** 10.3390/microorganisms9030654

**Published:** 2021-03-22

**Authors:** Giorgia Perpetuini, Alessio Pio Rossetti, Noemi Battistelli, Giuseppe Arfelli, Rosanna Tofalo

**Affiliations:** Faculty of Bioscience and Technology for Food, Agriculture and Environment, University of Teramo, Via R. Balzarini 1, 64100 Teramo, Italy; gperpetuini@unite.it (G.P.); alessiopio.rossetti@gmail.com (A.P.R.); nbattistelli@unite.it (N.B.); garfelli@unite.it (G.A.)

**Keywords:** yeasts, wine, adhesion properties, cleaning agents, MATs

## Abstract

In this study, yeasts isolated from filter membranes used for the quality control of bottled wines were identified and tested for their resistance to some cleaning agents and potassium metabisulphite, adhesion to polystyrene and stainless-steel surfaces, and formation of a thin round biofilm, referred to as a MAT. A total of 40 strains were identified by rRNA internal transcribed spacer (ITS) restriction analysis and sequence analysis of D1/D2 domain of 26S rRNA gene. Strains belong to *Pichia manshurica* (12), *Pichia kudriavzevii* (9), *Pichia membranifaciens* (1), *Candida sojae* (6), *Candida parapsilosis* (3), *Candida sonorensis* (1), *Lodderomyces elongisporus* (2), *Sporopachydermia lactativora* (3), and *Clavispora lusitaniae* (3) species. Regarding the adhesion properties, differences were observed among species. Yeasts preferred planktonic state when tested on polystyrene plates. On stainless-steel supports, adhered cells reached values of about 6 log CFU/mL. MAT structures were formed only by yeasts belonging to the *Pichia* genus. Yeast species showed different resistance to sanitizers, with peracetic acid being the most effective and active at low concentrations, with minimum inhibitory concentration (MIC) values ranging from 0.08% (*v/v*) to 1% (*v/v*). *C. parapsilosis* was the most sensible species. Data could be exploited to develop sustainable strategies to reduce wine contamination and establish tailored sanitizing procedures.

## 1. Introduction

The grapevine (*Vitis vinifera*) phyllosphere and wine musts harbor a complex microbiome, including yeasts, filamentous fungi, and bacteria that modulate grapevine health, growth, and wine fermentation [1,2]. During wine fermentation, these microbes interact among them during alcoholic (AF) and malolactic (MLF) fermentations. This dynamic interaction has great influence on the nutritional, hygienic, safety, and organoleptic characteristics of the final product. In particular, fermentative yeasts (e.g., *Saccharomyces cerevisiae*) and lactic acid bacteria (LAB, predominantly *Oenococcus oeni*) modulate the flavor and aroma of wine [3]. However, apart from *S. cerevisiae*, recognized as the main fermentative agent, other yeast species, known as non-*Saccharomyces* yeasts, such as *Hanseniaspora/Kloeckera*, *Pichia*, *Candida*, or *Metschnikowia*, release metabolites, which influence the chemical environment and/or fermentation process and are implicated in early stages of the AF [4]. These microorganisms could originate from the vineyard soil [5,6], air, precipitation (rainfall, hail, snow), be transported by animal vectors (bees, insects, and birds) [7,8,9], and be resident in nearby native forests [6,10]. Some of these wine-related non-*Saccharomyces* yeasts (e.g., *Brettanomyces bruxellensis*, *Pichia manshurica*) can spoil wine (e.g., volatile acidity and phenolic odors) [11,12]. Wine spoilage yeasts mainly belong to the following genera: *Dekkera/Brettanomyces*, *Candida, Hanseniaspora, Pichia, Metschnikowia, Saccharomycodes, Schizosaccharomyces*, and *Zygosaccharomyces* [13], which can cause film formation in bulk wines, cloudiness, sediment formation, and gas production in bottled wines, and off-flavor production during all processing and storing stages [14]. *Saccharomyces cerevisiae* can also be regarded as a spoilage microorganism, when associated with the refermentation of bottled wines [12,14]. According to previously published studies, the production environment harbors yeast strains, including spoilage ones, that are the most adapted to the processing environments and to the ecological niches. Moreover, the modes of contamination are often specific to each processing facility. Therefore, it is essential to study each manufacturing plant to determine the origin of the spoilage yeasts. The adhesion properties and biofilm-forming ability of spoilage yeasts have been recently reported for *B. bruxellensis* [15], and *P. manschurica* from organic wines [16]. The susceptibility of planktonic and biofilm yeast cells to different disinfectants and cleaning procedures were reported for *B. bruxellensis* [17], *Zygosaccharomyces rouxii* [18], and *Pichia pastoris* [19]. However, no data are available for other wine spoilage/contaminant yeasts. One of the main challenges of the wine industry is to identify new spoilage species and determine their physiological characteristics and ability to resist sanitization procedures. The financial burden generated by the use of ineffective and excess amounts of disinfectants may be reduced by choosing an appropriate disinfectant according to the dominant species of microorganism. Environmental pollution from the use of excessive quantities of chemicals may similarly be avoided. Therefore, in the present study, yeasts isolated from filter membranes used for the quality control of bottled wines were tested for their ability to adhere on abiotic surfaces and to form MAT structures (MATs). MATs are complex multicellular structures composed of yeast form cells [20]. The ability to form this kind of biofilm has been tested for some commercial wine yeast strains since it could suggest the ability of tested strains to adhere to surfaces of the wine environment, grape berries, and grapevine and to establish associations with other microbes, thereby affecting microbial dynamics during fermentation [21]. Finally, strains were tested for their resistance to potassium metabisulphite—since it shows a well-known anti-fungal activity and, together with membrane filtration, represents the main currently applied control action—and some cleaning agents. In particular, sodium hydroxide/sodium hypochlorite-based detergent and peracetic acid-based sanitizing agent were tested since they are generally used in cellars to reduce/prevent wine spoilage.

## 2. Materials and Methods

### 2.1. Origin of Samples

Yeasts were isolated from Biosart^®^ 100 Monitors filter membranes (0.45 μm) (Sartorius, Monza, Italy). These filters are generally used for the quality control of bottled wines. The samples were obtained from the following cultivars: Pinot, Cerasuolo, Passerina, Montepulciano, Pecorino, Trebbiano, Merlot Cabernet, and Merlot. All colonies present on the filters were isolated and streaked on YPD medium (yeast extract 1% *w/v*, peptone 2% *w*/*v*, glucose 2% *w*/*v*, and agar 2% *w*/*v*; Oxoid, Milan, Italy). Plates were incubated at 30 °C for 48 h. Colonies were purified by repetitive streaking passages in YPD medium. All strains were stored at −80 °C in YPD broth supplemented with glycerol (20% *v/v* final concentration) (Sigma-Aldrich Srl., Milan, Italy) or on YPD agar at 4 °C for short-term storage. They belong to the Culture Collection of Microbial Biotechnology Laboratory (Faculty of BioScience and Technology for Food, Agriculture and Environment, University of Teramo).

### 2.2. Yeasts Identification

Genomic DNAs were extracted using the InstaGene Matrix Kit (Bio-Rad, Milan, Italy) according to the manufacturer’s instructions. The 5.8S internal transcribed spacer (ITS) rRNA region was amplified in a Bio-Rad thermocycler (MyCycler, Bio-Rad) using primers ITS1 (5′ TCCGTAGGTGAACCTGCGG 3′) and ITS4 (5′ TCCTCCGCTTATTGATATGC 3′), as previously described [22]. Amplicons were digested with the following restriction enzymes: *Cfo*I, *Hae*III, and *Hinf*I [22]. Species identification was confirmed via sequencing D1/D2 domain of 26S rRNA gene, employing NL1 (5′- GCATATCAATAAGCGGAGGAAAAG -3′) and NL4 (5′- GGTCCGTGTTTCAAGACGG -3′) primer pairs [23]. Amplified fragments were purified using ExoSAP-IT Express PCR Cleanup (Thermo Fisher, Monza, Italy) according to the manufacturer’s instructions and sent to BMR Genomics (Padua University, Padua, Italy) for sequencing. The obtained sequences were compared to those available in the GenBank database (http://www.ncbi.nml.nih.gov/BLAST, accessed on 24 February 2021) and those of the Ribosomal Database Project (http://rdp.cme.msu.edu/index.jsp, accessed on 24 February 2021) to determine the closest known relative species on the basis of 26S rRNA gene homology [24].

### 2.3. Adhesion Properties of Yeasts

The adhesion properties of yeasts were tested on polystyrene and on stainless-steel surfaces. Yeasts were inoculated in six-well polystyrene plates in YPD medium and incubated at 30 °C for two weeks. Plate counts of planktonic and sessile cells were performed according to Perpetuini et al. [16]. Yeasts were also inoculated on stainless-steel supports according to Tomičić and Raspor [25]. Unattached cells were removed, while sessile cells were detached through several passages of rinsing and pipetting with saline solution (NaCl 0.85% *w/v*) and recovered. Viable count on YPD medium was carried out. All analyses were performed in triplicate only on viable and cultivable cells.

### 2.4. MAT Formation

The multicellular growth pattern (MAT formation) of yeast strains was tested as described by Reynold and Fink [20]. Yeast cells were inoculated in the center of YPD soft agar plates (agar 0.3% *w/v*) using a toothpick and incubated at 25 °C for 15 days. Plates containing 2% (*w/v*) agar were used as negative controls. The diameter was evaluated by photographing the plates and analyzing the photos with ImageJ software (http://imagej.nih.gov, accessed on 24 February 2021) according to Schneider et al. [26]. All tests were performed in triplicate.

### 2.5. Determination of the Minimum Inhibitory Concentrations (MICs)

Commercial cleaning agents commonly used for the hygienic practices in wineries as well as potassium metabisulphite were tested against the isolated yeasts. The agents were diluted to various concentrations within the in-use range: sodium hydroxide/sodium hypochlorite-based detergent (Enoclin Cloractiv L—SH) 0.05–5% (*v*/*v*) (L’Enotecnica, Nizza Monferrato, Italy); peracetic acid-based sanitizing agent (Enoclin Peracetic—PA) (L’Enotecnica) 0.02–10% (*v*/*v*); potassium metabisulphite 5–1600 ppm (L’Enotecnica). The minimum inhibitory concentrations (MICs) were evaluated according to Tristezza et al. [27]. Assays were performed in triplicate using the microtiter dilutions method. The MIC was defined as the lowest concentration of the disinfectant agent that prevents visible growth, by production of turbidity or pellet, after an incubation period of 48 h. *Pichia manshurica* PED 141-1 strain was used as control [16].

### 2.6. Statistical Analysis

Data were analyzed by means of Prism 7.0 program (GraphPad Software Inc., La Jolla, CA, USA) using *t*-test. A level of *p* < 0.05 was considered statistically significant.

## 3. Results and Discussion

### 3.1. Yeast Identification

A total of 40 yeasts were isolated from filter membranes. Restriction profile comparisons between isolates and published strains allowed assigning the isolates to *Pichia manshurica* (12), *Pichia kudriavzevii* (9), *Pichia membranifaciens* (1), *Candida sojae* (6), *Candida parapsilosis* (3), *Candida sonorensis* (1), *Sporopachydermia lactativora* (3), *Lodderomyces elongisporus* (2), and *Clavispora lusitaniae* (3) (Table S1). The D1/D2 domain of the 26S rRNA gene was sequenced and subsequently compared with sequences available in the GenBank database and the Ribosomal Database Project to confirm identification results. All the sequences obtained displayed similarity values ranging from 99% to 100% to reference sequences, confirming the identity of isolates (Table S1). *P. manshurica* is common in several fermented foods, and it is usually found in the early stages of spontaneous fermentation of alcoholic beverages, where it was firstly isolated [28]. It has also been found in spoiled and organic wines, suggesting that it is well adapted to wine environment and could be considered a contaminant in wineries [29]. This species is associated with off-flavors, off-odors [11], and biogenic amines production [30] and showed considerable hydrophobicity and biofilm formation on polystyrene [16]. *P. kudriavzevii* can be found in various fermented beverages (e.g., wine, beer, cereal-based beverage), soil, and fruits (e.g., mango pulp) [31,32]. It is a non-conventional yeast that is able to resist organic acids [33]. It can be considered a spoilage yeast in some food products, such as kimchi, where it is involved in the production of surface biofilm, off-odors, and texture softening [34,35]. Moreover, some strains isolated from nuruk—a Korean microbial starter for fermented products—showed high thermotolerance and ethanol production [36]. Among *Pichia* isolates, a strain of *P. membranifaciens* was also found. It can spoil wine and other fermented foods with the production of biofilm on the surface of wines and undesirable volatile compounds such as volatiles phenols [29,37]. It has also been previously studied for biofilm formation on stainless steel in filler implants in breweries [38]. Yeasts belonging to the *Candida* genus were identified as *C. sojae*, *C. parapsilosis*, and *C. sonorensis*. *C. sojae* and *C. parapsilosis* are placed in the *Lodderomyces–Spathaspora* clade [34] and are phylogenetically similar to *Lodderomyces elongisporus*, which was also found in the samples analyzed in this study. *C. sojae* was first isolated from effluents of a soybean extraction process [39] and it has a strong connection with *C. tropicalis* and *C. albicans* species, which are considered to be pathogenic, but differs from these species because of its inability to ferment maltose and because it has a low maximum growth temperature (below 40 °C). *C. parapsilosis* has been previously found in wine environments, probably originating from damaged grapes or soil [40,41]. A strain of *C. sonorensis* was found, probably brought by soil contamination because it is an asexual yeast species found only in the decaying tissue of cacti [42]. It is able to ferment glucose to ethanol, it is relatively tolerant to low pH environments, and has simple nutritional requirements [42,43]. Three strains of *Spor. lactativora* were isolated. This yeast was previously isolated from reverse osmosis filtration membranes, even after being subjected to a cleaning process, where it was able to form biofilm communities along with other filamentous fungi (*Magnusiomyces spicifer* and *Saprochaete clavata*) and Gram-negative bacteria [37,44]. Finally, three strains of *Cl. lusitaniae* were identified. This yeast has been previously isolated in grape and apple must, citrus fruits, and orange juice [45,46,47,48]. Species generally associated with wine spoilage such as *Brettanomyces*/*Dekkera*, *Zygosaccharomyces* spp., and *Saccharomyces* spp. were not identified [14].

### 3.2. Yeast Adhesion to Abiotic Surfaces

**Figure 1:**
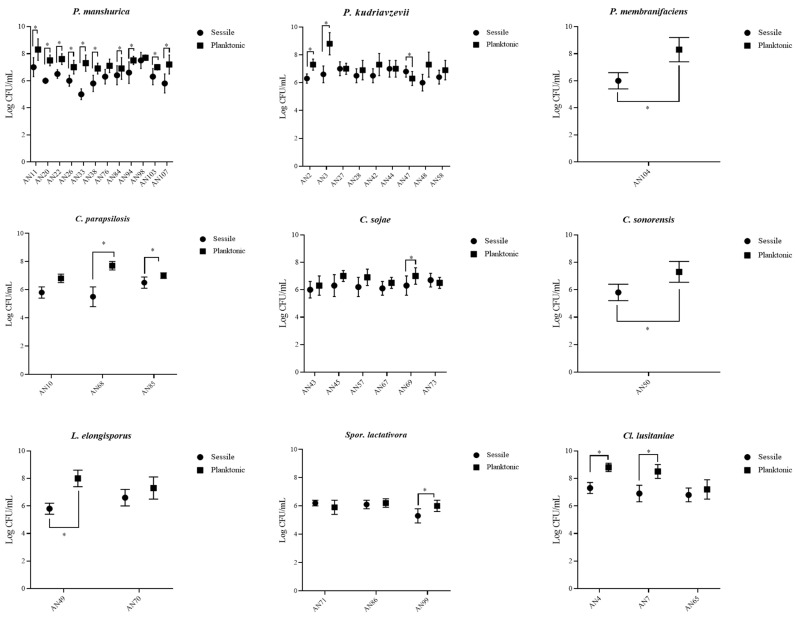
Viable yeast cell counts in planktonic and sessile states on polystyrene surface. * *p* < 0.05.

**Figure 2:**
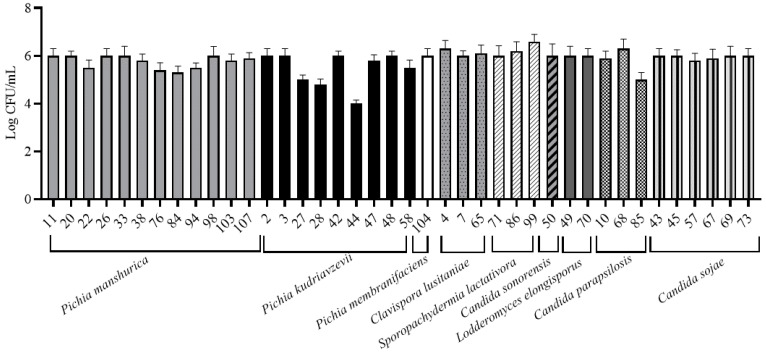
Viable yeast cell counts on steel surface.

Strains were tested for their ability to adhere on polystyrene and stainless-steel surfaces. The attention was focused on these materials since they are widespread in cellars (e.g., stainless-steel tanks, packaging for bottles, working surfaces, etc.). Differences were observed between species. On polystyrene plates, the planktonic state was favored (Figure 1). Intra- and inter-species differences were observed for planktonic and sessile cell counts. In general, planktonic cell counts were higher than sessile ones, with few exceptions (AN43, AN47, and AN71). For instance, *P. manshurica* sessile cells ranged from 5 log CFU/mL (AN33) to 7.5 log CFU/mL (AN98), while planktonic ones ranged from 6.9 log CFU/mL (AN84) to 8.3 log CFU/mL (AN11) (Figure 2). On stainless-steel surfaces, strains adhered with values of about 6 log CFU/mL. The lowest values were observed for three strains of *P. kudriavzevii* (AN27, AN28, and AN44) (Figure 2). Our data concerning *P. membranifaciens* are in agreement with Storgårds et al. [38], who showed that *P. membranifaciens*—as well as other yeast species such as *Candida krusei, Rhodotorula mucilaginosa, Wickerhamomyces anomalus* (ex *P. anomala*), and *S. cerevisiae*—formed biofilms on abiotic surfaces of brewery bottling plants. Biofilms of *P. membranifaciens* have been also reported in a rotating biological contactor (RBC) for the treatment of wine-cellar effluent [49]. Tomičić et al. [50] showed that yeasts can adhere to wooden surfaces. This ability is influenced by wood type, disinfectant, relative humidity, and temperature [50], but not by wood surface roughness [51]. The ability to form biofilm on wooden matrixes could represent a problem for wine quality since the spoilage metabolism of some yeast species such as *P. manshurica* could be maintained during wine aging [11]. The ability of *P. manshurica* to form biofilm on abiotic surfaces has been reported by our group and is mainly related to the hydrophobic nature of its surface [16]. However, it is important to underline that microbial attachment is often a two-step process. The first step is related to physico-chemical interactions, which are important to help the cells to approach the contact surface and attach loosely to it. The adhesion to abiotic surfaces is the first step of biofilm formation, and it is necessary to evaluate the biofilm-forming ability of these strains. Surface proteins/adhesins are involved in the second step since they support the cells to stick firmly to the surface [52]. Adhesion abilities have been described for the first time in *P. kudriavzevii* strains. As for the other members of the *Pichia* genus, this capacity is probably explained by their aerobic nature and fast growth [53]. The adhesion properties of *Spor. lactativora* have also been described by Vitzilaiou et al. [44]. These authors found that this species colonized the reverse osmosis membrane filtration elements from a whey water filtration unit. The majority of studies concerning the genus *Candida* have been performed on *C. albicans*. This species is able to colonize both abiotic and biotic surfaces through the expression of specific adhesins called Als [54]. Recently, Valotteau et al. [55] revealed the ability of *Candida glabrata* to adhere on abiotic surfaces thanks to Epa proteins (Epa1, Epa6, and Epa7), which contribute to both hydrophilic and hydrophobic interaction.

### 3.3. MATs Formation

MAT structures are considered an elaborate multicellular biofilm related to the sliding motility. According to Recht et al. [56], sliding motility is defined as a form of surface motility “produced by the expansive forces of the growing bacterial population in combination with cell surface properties that favour reduced friction between the cells and the substrate”. Only strains belonging to the *Pichia* genus were able to form MAT structures on semi-solid agar. MATs presented a central hub made of networks of cables and radial spokes with more or less jagged edges (Figure S1). Radial spokes were particularly evident in the MATs formed by *P. membranifaciens* AN104 strain. This structure was also less smooth and had more jagged edges than the others. All *P. kudriavzevii* strains as well as the *P. membranifaciens* strain showed structures covering all agar surface with a diameter of about 8.5 ± 0.44 cm, with the only exception being *P. kudriavzevii* AN3 strain, which had a diameter of 7.63 ± 0.32 cm. Intraspecies differences were especially detected for *P. manshurica* strains. The diameters ranged from 3.63 ± 0.45 cm (AN76) to 8.5 ± 0.61 cm (AN98). In particular, AN11, AN26, and AN33 strains showed values higher than 5 cm, while AN22 was 6.7 ± 0.38 cm, AN103 and AN94 were about 7 cm, AN76 and AN84 were more than 3 cm, while the others were about 8 cm.

MATs formation was previously described for *P. manshurica* by Perpetuini et al. [16], while it is reported for the first time in this study in *P. kudriavzevii* and *P. membranifaciens*. This phenomenon has been also described in other yeast species such as *Debaryomyces hansenii* and *Kluyveromyces marxianus* [57,58]. However, it is well characterized only in *S. cerevisiae* [20,59,60]. In this species, the ability to form biofilm and MAT structures is associated with the expression of specific adhesins such as Flo11p. Thirteen different structures of Flo11p have been described [61], and in some cases the Flo11 domain is present in double or triple copies, such as in some non-*Saccharomyces* species (e.g., *Kluyveromyces lactis, Cl. lusitaniae*, and *C. parapsilosis*) [62,63]. Moreover, Perpetuini et al. [58] showed that orthologs of *FLO*11 and *STE*12 genes were overexpressed in MAT structures formed by dairy *K. marxianus* strains. Some studies have indicated that in *Candida* spp. many *ALS* genes are regulated by orthologues of the pathways known to regulate adhesion in *S. cerevisiae* [64,65,66,67]. Moreover, in *C. albicans*, six transcriptional regulators (*Efg1, Tec1, Bcr1, Ndt80, Brg1,* and *Rob1*) involved in biofilm formation have been described [68], while in *C. glabrata*, lectin-like adhesins include *EPA* gene products [69]. However, the presence of these adhesins is not sufficient to explain MAT structures formation. This phenomenon appears to be quite complex and related to strain ploidy: increased ploidy reduced MATs formation, with a tetraploid strain showing almost no MAT formation [20]. Moreover, additional pathways acting in a Flo11-independent manner have been described. Sarode et al. [70] described this kind of pathway, referring to it as the biofilm pathway. It involves the class E vacuolar protein sorting (vps) components of the multivesicular body pathway. In 2014, the same authors identified a cell wall signaling protein (Wsc1p) impacting MATs formation [60]. Obtained data suggest that the *Pichia* genus could probably present in its genome specific adhesins and tailored metabolic pathways involved in MATs formation.

### 3.4. Determination of the Minimum Inhibitory Concentrations (MICs)

The main source of microbial contamination during food production may be the processing plant itself, caused by unsuccessful hygiene measures, the ability of yeasts to withstand a stressful environment, and/or the inefficacy of disinfectants [71]. Therefore, it is important to identify new contaminant/spoilage species and determine their physiological characteristics and ability to resist sanitization procedures. For this reason, strains were tested for their resistance to PA, SH, and potassium metabisulphite. The MICs are reported in Table 1. Yeast species showed MIC differences, suggesting that the resistance is species-dependent, and strains within the species had different behaviors. Similar results have been reported in other studies [72,73,74]. PA was the most effective sanitizer, being active at low concentrations. In fact, MIC values ranged from 0.08% to 1%, with *C. parapsilosis* having the most sensible strains. SH detergent was more effective at lower concentrations than the recommended in-use concentration. In fact, strains showed MICs ranging from 0.4% to 2%. Regarding potassium metabisulphite, MICs varied from 80 ppm to 160 ppm. *Spor. lactativora* was the most sensitive species to the tested compounds, exhibiting the lowest MIC, followed by *L. elongisporus*, *Cl. Lusitaniae*, and *C. parapsilosis*, which were sensitive to PA, but slightly resistant to SH and potassium metabisulphite. *Pichia* spp. showed the highest resistance to potassium metabisulphite and presented similar MIC values for PA and SH compared to other species. *C. sojae* and *C. sonorensis* exhibited good resistance to SH but limited resistance to PA and potassium metabisulphite. PA and SH detergents were effective against all species tested, which is in agreement with previous studies [16,27]. *P. manshurica* strains had a resistance to PA similar to values reported by Perpetuini et al. [16] with only two strains (33 and 76) showing a higher resistance. Obtained MICs for SH were lower than those reported by the same authors. In fact, a strain showed values of MIC of 5%, which is equal to the maximum recommended in-use concentration, while the other strains presented values ranging from 1% to 3%. Winniczuk and Parrish [75] reported the efficacy of hypochlorite, peracetic acid, phosphoric acid, and anionic compounds against yeast strains isolated from orange juice. The efficiency of potassium metabisulphite is species- and strain-dependent and can produce growth retardation or cell killing [76]. As expected, the values of MIC were low, which is in agreement with other authors [16,77].

## 4. Conclusions

The selective pressure exerted by the environment during wine production reduces the natural yeast diversity to a limited number of well-adapted species. However, contaminant yeasts can develop, and an understanding of the origin and routes of contamination of these species can contribute to a more effective control of production processes. This study revealed novel information on the diversity and preservative resistance of yeasts encountered in the wine environment. A strategy to control yeast contamination should be the examination of the production lines to identify possible points in the processes where wines could be exposed to yeast contamination. Further studies will be focused on *P. manshurica* in order to better characterize its ability to form biofilm using different media (e.g., wine) and to identify the genes involved in its adhesion. This information will be useful to establish a link between the genomic background and phenotypic traits of adherent spoilage yeasts. Additional studies are also necessary to evaluate the susceptibility of yeasts to common and novel disinfectants in the planktonic and biofilm state. A close examination of the biofilm-forming capacity of strains will be performed. In fact, the application of disinfectants in the winery is important to remove the adhered cells in order to prevent biofilms formation since they are difficult to eradicate. These results could be useful to develop new strategies to decrease wine contamination and to stress the importance of accurate sanitizing procedures designed ad hoc to eradicate resistant populations. This kind of study is useful to develop new strategies to eliminate/reduce wine contamination and/or control yeast growth and thus reduce food waste. Moreover, better knowledge about contaminant yeasts provides a fundamental tool to implement and improve the HACCP (Hazard Analysis and Critical Control Points) systems.

## Figures and Tables

**Table 1:** Minimum inhibitory concentrations (MICs) of cleaning agents and potassium metabisulphite.

Strains	Species	PA (0.05–10%)	SH (0.5–5%)	Potassium Metabisulphite (5–1600 ppm)
AN11	*P. manshurica*	0.2	1	140
AN20, AN22, AN38		0.2	1.2	160
AN26		0.2	1.2	140
AN33, AN76		0.4	1.2	160
AN84, AN103		0.2	1.2	150
AN94		0.2	1	160
AN98, AN107		0.2	1.2	160
PED 141-1		0.25	5	150
AN2, AN3	*P. kudriavzevii*	1	2	160
AN27, AN28		0.8	1.2	120
AN42		0.6	1.2	140
AN44		0.6	1.2	80
AN47, AN48, AN58		1	2	160
AN4	*Cl. lusitaniae*	1	2	120
AN7		0.5	1.2	100
AN65		1	2	130
AN71	*Spor. lactativora*	0.2	0.8	120
AN86		0.5	0.4	80
AN99		0.1	0.6	100
AN10, AN68, AN85	*C. parapsilosis*	0.08	1	120
AN43		0.1	2	130
AN45		0.1	2	120
AN57	*C. sojae*	0.2	2	110
AN67, AN73		0.8	2	80
AN69		1	2	120
AN104	*P. membranifaciens*	0.6	1.5	140
AN50	*C. sonorensis*	1	2	130
AN49, AN70	*L. elongisporus*	0.6	1.5	100

## Data Availability

Data is contained within the article or Supplementary Material.

## Supplementary Materials

The following are available online at https://www.mdpi.com/2076-2607/9/3/654/s1: Table S1: Yeasts origin and identification. Figure S1: MATs formation on soft agar by tested strains at 25 °C after 7 days.
