# Supplementary material for: Adhesion Properties, Biofilm Forming Potential, and Susceptibility to Disinfectants of Contaminant Wine Yeasts
Source: Microorganisms. 2021 Mar 22;9(3):654. doi: 10.3390/microorganisms9030654 (PMC8004283; doi:10.3390/microorganisms9030654)
Supplement: Supplementary file 1 [file microorganisms-09-00654-s001.zip › Supplementary file/Table S1. Yeasts origin and identification .docx]

**Table S1.** Yeasts origin and identification based on PCR-RFLP of 5.8S ITS gene and sequencing of 26S rRNA gene.

| ID | ORIGIN | SPECIES | ITS (bp) | *HAE* III (bp) | *HINF* I (bp) | *CFO* I (bp) | ACC. NUMB. |
| --- | --- | --- | --- | --- | --- | --- | --- |
| AN1, AN20, AN22, AN33 | Pinot Gris | *P. manshurica* | 460 | 5 + 10 + 50 + 95 + 300 | 200 + 260 | 60 + 70 + 100 + 230 | MH169586.1 |
| AN26 | Cerasuolo |  |  |  |  |  |  |
| AN38 | Passerina |  |  |  |  |  |  |
| AN76, AN84 | Montepulciano d’Abruzzo |  |  |  |  |  |  |
| AN94 | Pecorino |  |  |  |  |  |  |
| AN98 | Trebbiano |  |  |  |  |  |  |
| AN103, AN107 | Pecorino Sparkling |  |  |  |  |  |  |
| AN2, AN3 | Montepulciano d’Abruzzo | *P. kudriavzevii* | 500 | 40 + 100 + 380 | 130 + 160 + 210 | 60 + 70 + 180 + 210 | MT151655.1 |
| AN27, AN28, AN48 | Pecorino |  |  |  |  |  |  |
| AN42 | Merlot |  |  |  |  |  |  |
| AN44 | Merlot Cabernet Sauvignon |  |  |  |  |  |  |
| AN47, AN58 | Trebbiano |  |  |  |  |  |  |
| AN104 | Pecorino Sparkling | *P. membranifaciens* | 480 | 10 + 50 + 90 + 330 | 200 + 280 | 60 + 70 + 100 + 250 | MK358179.1 |
| AN4, AN65 | Montepulciano d’Abruzzo | *Cl. lusitaniae* | 370 | 370 | 180+160 | 210+180 | MT334451.1 |
| AN7 | Cerasuolo |  |  |  |  |  |  |
| AN71 | Montepulciano d’Abruzzo | *Spor. lactativora* | 600 | 600 | 260 + 240 | 600 | KY109770.1 |
| AN86 | Cerasuolo |  |  |  |  |  |  |
| AN99 | Trebbiano |  |  |  |  |  |  |
| AN50 | Montepulciano d’Abruzzo | *C. sonorensis* | 750 | 200 + 550 | 320 + 420 | 290 + 360 | KY106769.1 |
| AN49, AN70 | Montepulciano d’Abruzzo | *L. elongisporus* | 600 | 600 | 270 + 275 | 250 + 330 | MN700893.1 |
| AN10 | Trebbiano | *C. parapsilosis* | 550 | 110 + 420 | 260 + 280 | 240 + 300 | MT001266.1 |
| AN68, AN85 | Cerasuolo |  |  |  |  |  |  |
| AN43, AN45, AN73 | Merlot Cabernet Sauvignon | *C. sojae* | 550 | 70 + 450 | 270 + 275 | 260 + 280 | KY106762.1 |
| AN57, AN67, AN69 | Cerasuolo |  |  |  |  |  |  |
