# Supplementary figures and images for: Adhesion Properties, Biofilm Forming Potential, and Susceptibility to Disinfectants of Contaminant Wine Yeasts
Source: Microorganisms. 2021 Mar 22;9(3):654. doi: 10.3390/microorganisms9030654 (PMC8004283; doi:10.3390/microorganisms9030654)

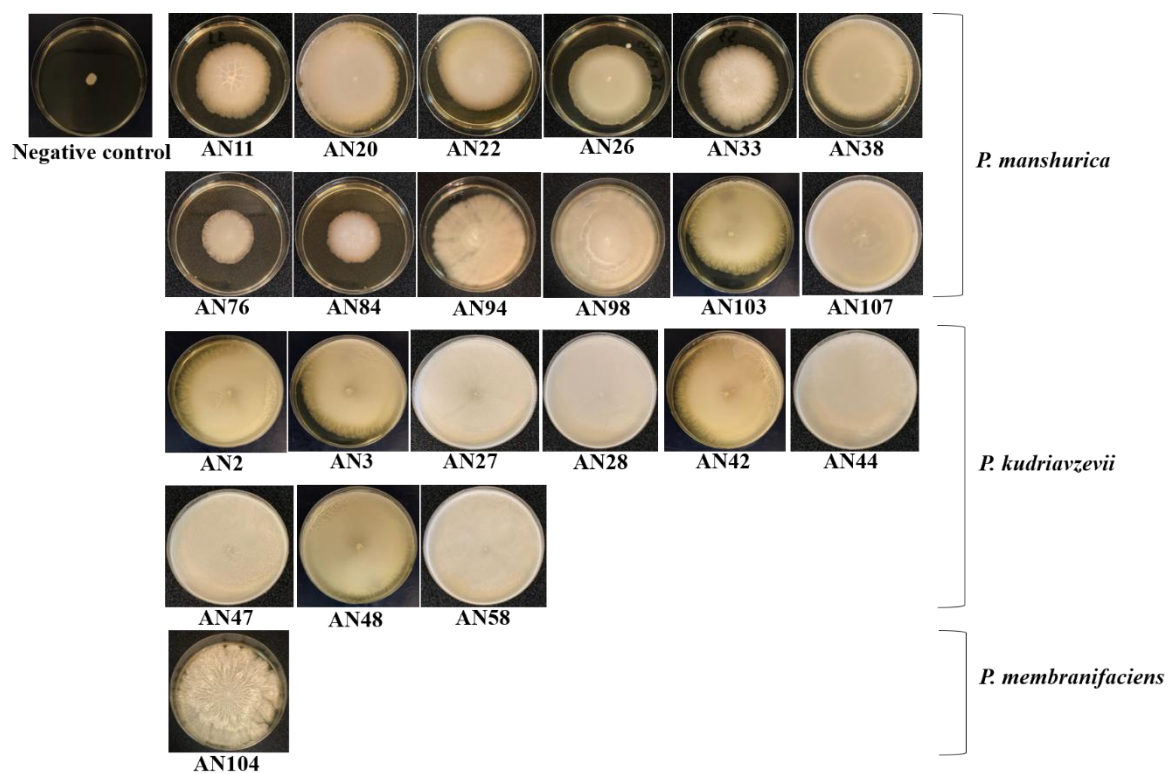

Figure S1

Supplement: Supplementary file 1 [file microorganisms-09-00654-s001.zip › Supplementary file/Figure S1.pdf]
